# Shedding metabo‘light’ on the search for sepsis biomarkers

**DOI:** 10.1186/s13054-015-0969-7

**Published:** 2015-07-07

**Authors:** Claudia C. dos Santos

**Affiliations:** Interdepartmental Division of Critical Care, The Keenan Research Centre of the Li Ka Shing Knowledge Institute of St Michael’s Hospital, 30 Bond Street, Toronto, ON M5B 1W8 Canada

## Abstract

The clinical presentation of severe infection with generalized inflammation is similar, if not identical, to systemic inflammation induced by sterile tissue injury. Novel models and unbiased technologies are urgently needed for biomarker identification and disease profiling in sepsis. Here we briefly review the article of Kamisoglu and colleagues in this issue of *Critical Care* on comparing metabolomics data from different studies to assess whether responses elicited by endotoxin recapitulate, at least in part, those seen in clinical sepsis.

Our inability to differentiate sepsis from non-infectious inflammatory states has negatively impacted research developments in diagnosis, prognosis and treatment of sepsis. Compounding the problem, biomarker identification and disease profiling is hampered by our reliance on a theoretical construct that assumes disseminated infection stimulates pattern recognition receptors, such as Toll like receptor (TLR)4 in response to lipopolysaccharide, to generate clinically recognizable, biochemically defined common pathways of response in the host. The same mediators that cause general inflammation and harm in sepsis are required for host defence, numerous pathways are highly redundant, and receptors that distinguish self from non-self are also needed for the recognition of danger signals. The result is that many of the biomarkers used in the ICU are neither sensitive nor specific enough to inform regarding specific pathophysiologic processes.

Recent advances in ‘omic’ technologies have opened new opportunities for sepsis research. In a recent article published in *Critical Care*, Kamisoglu and colleagues [1] used metabolomics to assess whether responses elicited by endotoxin recapitulate, at least in part, those seen in clinical sepsis [2]. The study is primarily a retrospective *in silico* analysis of metabolomes obtained from subjects who participated in an experimental endotoxemia study [3] and from patients enrolled in the Community Acquired Pneumonia and Sepsis Outcome and Diagnostics (CAPSOD) study who after independent audit fulfilled criteria for sepsis and outcomes [4]. Patients in the CAPSOD cohort were classified as uncomplicated sepsis, severe sepsis, septic shock, and non-infected systemic inflammatory response syndrome (‘ill’ controls with non-infectious SIRS). Metabolic profiling of plasma in both studies was performed using non-targeted mass spectrometry by the same commercial provider. In contrast to targeted approaches that profile a small number of known metabolites, untargeted approaches (without restriction to particular compounds) have the advantage of allowing for identification of metabolic fingerprints (that is, multiple biomarkers that form a biopattern) associated with particular endophenotypes [5, 6]. The important insights of the study are two: the clinical relevance of endotoxemia in sepsis, and the applicability of metabolomics as an analytical tool in sepsis. Because lipopolysaccharide acts through the TLR4, endotoxin challenge is a model of TLR4 agonist-induced SIRS [2]. The issue of the contribution of TLR signaling in sepsis will be difficult to unravel as these receptors are likely to be activated by both primary (pathogen-related) and secondary (host-related) events. A discussion of the merits and limitations of comparing high throughput data from different studies is fundamental to the use of genomic technologies in critical illness.

Metabolomics is heavily supported by mass spectrometry (MS) and nuclear magnetic resonance (NMR) as parallel technologies that provide an overview of the complete set of small-molecule chemicals found within a biological sample (metabolome) [4]. The main advantage of MS is sensitivity - it can detect analytes routinely in the femtomolar to attomolar range. Coupling MS with separation techniques - liquid chromatography (LC) or gas chromatography (GC) - enhances the detection ability of MS. The major weakness of MS is quantification. In contrast, in NMR the peak area of a compound is directly related to the concentration of specific nuclei, making quantification very precise. NMR is, however, much less sensitive. Therefore, a single analytical tool is unlikely to detect all possible metabolites, suggesting a combination of techniques will be required to assign metabolites and patients to specific classes [3]. Moreover, of all the systems biology disciplines, metabolomics is closest to the phenotype, is profoundly affected by environmental factors, and dynamic changes suggest selection of appropriate time points for biomarker identification will be critical [4, 7].

In the study by Kamisoglu and colleagues biochemical profiles obtained by GC-MS and LC-tandem MS provided information on a total of 366 metabolites. Since no significant differences were identified for plasma metabolites between subgroups of sepsis survivors, different groups were collapsed into a single group. While loss of resolution (small number of metabolites), and discordant time points, makes it difficult to classify clinically relevant endophenotypes, the approach selected by Kamisoglu and colleagues to pool ‘similar’ patients into a single group is extensively used in systems biology to increase the statistical power to detect differences between groups [8, 9]. Cluster analysis, pooling metabolite groups, also enhances the likelihood of finding clinically relevant class-specific signatures [10, 11]. Metabolic data from sepsis survivors and non-survivors were also pooled to compare patients with sepsis and non-infected SIRS. Despite individual variability, the metabolic responses to endotoxin are similar to those seen in sepsis survivors. The authors rationalized that similar metabolomes may reflect TLR4 agonist-induced SIRS or common processes of recovery. They were also able to identify specific features that differentiate patients with SIRS from both endotoxin and sepsis patients. While one of the strengths of this study is the combination of clinical data, severity assignment and metabolomics, an important limitation is in the assumption that absence of detectable differences indicates groups are comparable. Also, restricting the intra-study comparative analyses to metabolites that significantly change between different conditions maximizes the likelihood of detecting overall correlations between studies [9].

After stratification of sepsis patients based on 28-day survival, the direction of change of 21 of 23 metabolites was the same in endotoxemia and sepsis survivors (compared with non-survivors). Similar to other studies, the metabolite group that differentiated surviving versus non-surviving CAPSOD patients was acylcarnitines [12]. In the study by Kamisoglu and colleagues, comparison between studies was possible because the proprietary extraction protocol used for sample preparation was the same in both studies - minimizing the variability associated with sample preparation. Standard procedures consistent across labs will be required if we want to deposit and compare raw (meta)data across studies. This is the goal of the human metabolome project, which aims to identify, quantify, and catalogue all metabolites in human tissues and biofluids [13]. In the future, integration networks using different types of ‘omic’ data will be combined to allow more thorough and comprehensive modeling of complex traits [14]. Overall, studies such as the one conducted by Kamisoglu and colleagues are pioneering in that they are setting the precedent for how will we integrate, compare, analyze and generalize results from high throughput technologies.

## Abbreviations

CAPSOD: Community acquired pneumonia and sepsis outcome and diagnostics
GC: Gas chromatography
LC: Liquid chromatography
MS: Mass spectrometry
NMR: Nuclear magnetic resonance
SIRS: Systemic inflammatory response syndrome
TLR: Toll like receptor

## References

[1] Kamisoglu K, Haimovich B, Calvano SE, Coyle SM, Corbett SA, Langley RJ (2015). Human metabolic response to systemic inflammation: assessment of the concordance between experimental endotoxemia and clinical cases of sepsis/SIRS. Crit Care.

[2] Artenstein AW, Higgins TL, Opal SM (2013). Sepsis and scientific revolutions. Crit Care Med.

[3] Banoei MM, Donnelly SJ, Mickiewicz B, Weljie A, Vogel HJ, Winston BW (2014). Metabolomics in critical care medicine: a new approach to biomarker discovery. Clin Invest Med.

[4] Sriyudthsak K, Shiraishi F, Hirai MY (2013). Identification of a metabolic reaction network from time-series data of metabolite concentrations. PLoS One.

[5] Lacy P (2011). Metabolomics of sepsis-induced acute lung injury: a new approach for biomarkers. Am J Physiol Lung Cell Mol Physiol.

[6] Kaddurah-Daouk R, Kristal BS, Weinshilboum RM (2008). Metabolomics: a global biochemical approach to drug response and disease. Annu Rev Pharmacol Toxicol.

[7] Zheng Y, Yu B, Alexander D, Mosley TH, Heiss G, Nettleton JA, Boerwinkle E (2013). Metabolomics and incident hypertension among blacks: the atherosclerosis risk in communities study. Hypertension.

[8] Arakawa K, Tomita M (2013). Merging multiple omics datasets in silico: statistical analyses and data interpretation. Methods Mol Biol.

[9] Bartel J, Krumsiek J, Theis FJ (2013). Statistical methods for the analysis of high-throughput metabolomics data. Comput Struct Biotechnol J.

[10] Oresic M, Hyotylainen T, Herukka SK, Sysi-Aho M, Mattila I, Seppanan-Laakso T (2011). Metabolome in progression to Alzheimer’s disease. Transl Psychiatry.

[11] Bowling FG, Thomas M (2014). Analyzing the metabolome. Methods Mol Biol.

[12] Schmerler D, Neugebauer S, Ludewig K, Bremer-Streck S, Brunkhorst FM, Kiehntopf M (2012). Targeted metabolomics for discrimination of systemic inflammatory disorders in critically ill patients. J Lipid Res.

[13] The Human Metabolome Project (HMDB). http://www.hmdb.ca/. Accessed 18 May 2015.

[14] Yoon SH, Han MJ, Jeong H, Lee CH, Xia XX, Lee DH (2012). Comparative multi-omics systems analysis of Escherichia coli strains B and K-12. Genome Biol.

